# Perceived need for treatment and non-utilization of outpatient psychotherapy in old age: two cohorts of a nationwide survey

**DOI:** 10.1186/s12913-021-06384-6

**Published:** 2021-05-10

**Authors:** Paul Gellert, Sonia Lech, Eva-Marie Kessler, Wolfram Herrmann, Susanne Döpfmer, Klaus Balke, Monika Oedekoven, Adelheid Kuhlmey, Susanne Schnitzer

**Affiliations:** 1 Charité – Universitätsmedizin Berlin, Institute of Medical Sociology and Rehabilitation Science, Charitéplatz 1, 10117 Berlin, Germany; 2 MSB Medical School Berlin, Department of Psychology, Working Unit Geropsychology, Berlin, Germany; 3 Charité – Universitätsmedizin Berlin, Institute of General Practice, Berlin, Germany; 4 German National Association of Statutory Health Insurance Physicians (Kassenärztliche Bundesvereinigung KBV), Berlin, Germany

**Keywords:** Psychotherapy, Healthcare utilization, Andersen’s model of health service use, Unmet need for treatment, Perceived need for treatment, Old age

## Abstract

**Beackground:**

Older adults with mental health problems may benefit from psychotherapy; however, their perceived need for treatment in relation to rates of non-utilization of outpatient psychotherapy as well as the predisposing, enabling, and need factors proposed by Andersen’s Model of Health Care Utilization that account for these differences warrant further investigation.

**Methods:**

We used two separate cohorts (2014 and 2019) of a weighted nationwide telephone survey in Germany of German-speaking adults with *N* = 12,197 participants. Across the two cohorts, 12.9% (weighted) reported a perceived need for treatment for mental health problems and were selected for further analyses. Logistic Generalized Estimation Equations (GEE) was applied to model the associations between disposing (age, gender, single habiting, rural residency, general health status), enabling (education, general practitioner visit) non-utilization of psychotherapy (outcome) across cohorts in those with a need for treatment (need factor).

**Results:**

In 2014, 11.8% of 6087 participants reported a perceived need for treatment due to mental health problems. In 2016, the prevalence increased significantly to 14.0% of 6110 participants. Of those who reported a perceived need for treatment, 36.4% in 2014 and 36.9%in 2019 did not see a psychotherapist – where rates of non-utilization of psychotherapy were vastly higher in the oldest age category (59.3/52.5%; 75+) than in the youngest (29.1/10.7%; aged 18–25). Concerning factors associated with non-utilization, multivariate findings indicated participation in the cohort of 2014 (OR 0.94), older age (55–64 OR 1.02, 65–74 OR 1.47, 75+ OR 4.76), male gender (OR 0.83), lower educational status (OR 0.84), rural residency (OR 1.38), single habiting (OR 1.37), and seeing a GP (OR 1.39) to be related with non-utilization of psychotherapy; general health status was not significantly associated with non-utilization when GP contact was included in the model.

**Conclusion:**

There is a strong age effect in terms of non-utilization of outpatient psychotherapy. Individual characteristics of both healthcare professionals and patients and structural barriers may add to this picture. Effective strategies to increase psychotherapy rates in those older adults with unmet treatment needs are required.

**Supplementary Information:**

The online version contains supplementary material available at 10.1186/s12913-021-06384-6.

## Introduction

The World Health Organisation (WHO) estimates that more than 20 % of adults aged sixty and over face mental health problems [1]. There is clear evidence that older adults respond equally well to psychotherapeutic and psychological treatment for mental health disorders, including depression compared to younger people (e.g., [2, 3]). Treatment response is unrelated to age itself, and potential limited responses are related to neurocognitive disorders and/or frailty [2, 3]. While depression is the most common across all age groups, depression in late life poses specific challenges to the individual and the healthcare system [4]. Symptoms of depression in old age may differ from younger cohorts, showing more vegetative and somatic rather than affective symptoms; moreover, comorbidities and associated loss of physical functioning, cognitive impairment, and chronic pain may overlap with depressive symptoms [5, 6]. Specific aspects such as loneliness, the loss of a spouse, and loss of physical functioning that may lead to care dependency are risk factors for developing mental health problems in old age [7]. Finally, negative age stereotypes may prevent patients and healthcare professionals from detecting depression adequately [8, 9]. However, there seems to be a large discrepancy in the proportion of older adults in need of psychological services and the low proportion that actually receives them. Despite recent studies are sparse, older adults have been shown to disproportionately underutilize professional mental health services relative to younger adults (e.g., [10–12]). This trend tends to be stable over time, while other studies report an increase (for a recent discussion, see [13]). For example, Byers et al. found that approximately 70 % of older adults with mood and anxiety disorders did not use mental health services [14]. Yet, determinants of the mental health treatment gap among older adults remain unclear, especially for German older adults. A recently published systematic review on barriers preventing older adults from seeking and accessing mental health care in the United States [15] found intrinsic hurdles, such as negative attitudes toward mental health care and lack of perceived need for treatment as main barriers.

Patients’ needs meet the professional health care system by utilizing health services. According to Andersen’s Model of Health Service Use [16], the utilization of health services is determined by three major components: *predisposing, enabling,* and *need factors*. *Individual predisposing factors* associated with non-utilization of mental health services among older adults include male gender and lower educational status [17] as well as age [10, 11]. With regard to *enabling factors*, the role of financial, marital, or cohabiting status as well as region of origin in the underutilization of mental health services among older adults remains inconsistent [14, 18] and need to be further investigated. Structural factors such as costs [19] and access to care [16] may be related to the non-utilization of outpatient mental health services among older adults. For example, in Germany, general practitioners (GPs) are often the first point of contact and play a key role in the referral of patients, and older adult patients in particular, with depressive symptoms to psychotherapeutic treatment [20]. However, previous empirical work has reported on deficient outpatient care for depressed individuals in Germany and diagnostic and treatment-related challenges of depression in old age in the primary care setting [8]. The literature describes the clinician’s decision to refer a patient with depression to a mental health specialist as complex, involving myriad aspects on a clinician, patient, and practice level [21]. It is of great interest to gain a better understanding of the role of GPs in the utilization of mental health services among older adults. Perceived need for treatment reflects the extent to which individuals are aware their mental health problems require professional help. These *need factors* may facilitate the utilization of mental health services [22]. Further, individuals’ views and experience of their own general health and their functional state were found to be associated with mental health care utilization [14]. The present study aims to examine factors contributing to the gap between the perceived need for treatment and non-utilization of psychotherapy in Germany. To our knowledge, no empirical data exists on trends and changes in perceived need for mental health treatment. However, positive changes related to stigma and mental health treatment have been reported in the past [23]. Drawing on these results, it is conceivable that the perceived need for mental health treatment increases with higher mental health prevalence.

### Aim of the study

The present study aimed to examine predisposing, enabling, and need factors of mental health service utilization across age groups by using data from two separate cohorts from 2014 and 2019. Based on the literature review and Andersen’s Model of Health Services Use, the following hypotheses are proposed: We expect there to be a positive trend of perceived need for treatment from 2014 to 2019 (Hypothesis 1). Furthermore, we hypothesize that the relative proportion of people with a perceived need for treatment due to a mental health problem who do not consult a psychotherapist is higher in older age groups than in younger ones (Hypothesis 2). Beyond older age, we expect male gender, low educational status, single habitation, rural residency, and poor general health status negatively associated with non-utilization of psychotherapy (Hypothesis 3). Finally, we expect the association of psychotherapy non-utilization with GP visits to be positive (i.e., acting as a facilitator), though previous evidence is mixed (Hypothesis 4).

## Methods

### Sample and procedure

We made use of two separate cohorts from 2014 and 2019 of the survey of the National Association of Statutory Health Insurance Physicians (Kassenärztliche Bundesvereinigung; KBV), which was planned and implemented in cooperation with the Charité – Universitätsmedizin Berlin and the research company Forschungsgruppe Wahlen Telefonfeld GmbH (FGW). The aim of the surveys in 2014 and 2019 was to record the current outpatient care situation in Germany. From April to May 2014 and March to April 2019, just over 6000 randomly selected citizens (*N =* 6087 in 2014; *N =* 6110 in 2019) in Germany were interviewed by telephone. The sample was drawn from the German-speaking resident population aged 18 and over. For the sample, a regionally stratified, two-step random sample was used. The data was weighted for gender, age, and education according to their nominal distributions across the adult population in Germany [24]. As there are no official statistics for gender, age, and education for German-speaking foreigners, they were assigned the weight 1. Taking into account the probabilistic foundations of random samples, the weighted survey can be considered as representative of the resident population of Germany aged 18 and over [24]. In total, *n* = 804 persons in 2014 and *n* = 862 in 2019 (*N* = 1666 unweighted; subsequently, results were reported for weighted analyses) reported distressing mental health problems and constituted the final sample.

### Measures

#### Psychotherapy non-utilization (outcome)

Those individuals who reported having had a distressing mental health problem that needed medical or psychological help were then asked if they had actually consulted a psychotherapist to address the problem.

#### Perceived need for treatment (need factor)

In order to define the number of people who reported perceived need for treatment for mental health problems, the following question was formulated: ‘In the past three years, have you had such a stressful emotional problem that you needed medical or psychological help?’ This item is adapted from the Perceived Need for Care Questionnaire [19].

#### Age and gender (predisposing factor)

Age was asked in categories of 5 years (the last category was aged 80 and older; 7.3% of participants across both cohorts fell into this category) and was re-categorized into 18–24, 25–34, 35–44, 45–54, 55–64, 65–74, 75+. Gender was coded 1 = women, 0 = men.

#### Single habiting (predisposing factor)

‘How many people live in your household in total?’ Those that replied ‘none’ were categorized as single habiting.

#### Rural residency (predisposing factor)

Participants were asked, ‘Approximately how many inhabitants does your town have?’ Answers under 5000 inhabitants were coded as living in a rural area.

#### Educational level (enabling factor)

Education has been operationalized according to the ISCED (International Classification of Education) category using a 3-step educational concept. In Germany, after 4 years of primary education, students are selected into one of the three secondary school tracks corresponding to 9th Grade (low), 10th Grade (middle), or 12th Grade (high). For post-school education, we dummy coded educational attainment into university degree yes/no.

#### General health status (enabling factor)

Participants were asked ‘How would you describe your state of health in the last four weeks?’ This item has been adapted from the general health single-item of the SF-36 scale [25], but a four-week time interval has been added. Answers were given on a 5-point scale: ‘excellent’, ‘very good’, ‘good’, ‘not good’, and ‘poor’. For analyses, ‘not good’ and ‘poor’ were considered poor health status.

#### GP visit (enabling factor)

GP visits were assessed by asking, ‘Have you been to a doctor’s office in the last 12 months?’ If participants indicated ‘yes’, it was then asked, ‘Was this a general practitioner?’

### Statistical analysis

Our dependent variable was the non-utilization of psychotherapy in those who perceived a need for treatment due to a mental health problem. For univariate comparisons between cohorts, chi-square tests were used. The hypotheses were tested applying generalized estimating equations (GEEs). GEEs represent an extension of the generalized linear models for correlated data (i.e., cohort cluster structure) and were specified for the binary dependent variable (i.e., not using psychotherapy = 1). Generalized Estimation Equation (GEE) models were specified (weighting factor was used; distribution = binomial; link function = logit; repeated = cohort; type of covariance = exchangeable; robust estimator was used). The weighting procedure used reference values of gender, age, and education, according to their nominal distributions across the adult population in Germany [24]. In the first step of the analysis, age (categorical: 18–24, 25–34, 35–44, 45–54, 55–64, 65–74, 75+) and cohort (2014 = 0, 2019 = 1; referring to Hypothesis 1) were included in the model as an independent variable to model the association of age and non-utilization of psychotherapy (Hypothesis 2). In the second model, additional determinants (Hypotheses 3), i.e., education (categorical), single habiting (1 = yes; 0 = no), rural residency (1 = yes; 0 = no), poor health status (1 = yes; 0 = no), and GP visits (Hypothesis 4; 1 = yes; 0 = no) were included to analyse the associations with non-use of psychotherapy. The statistical analyses were performed using the statistical software SPSS v25, and the significance level was set at 5% (*p* < .05).

## Results

In total, *N* = 12,197 participants (*n* = 6087 in 2014; *n* = 6110 in 2019) were included in the cohorts of the survey (Table 1). Of those, 52.6% (weighted percentages; thus no N is provided) were women and 7.6% were between 18 and 24, 11.8% 25–34, 14.1% 35–44, 19.7% 45–54, 18.4% 55–64, 14.6% 65–74, and 13.8% 75+ years of age. Concerning educational attainment, 34.8% had low education while 16.7% had a university degree. Furthermore, 17.9% were single habiting and 31.9% were living in a rural area. There were some differences across cohorts; there were more older adults (those aged 65–74 made up 4.0% in 2014, 15.5% in 2019; 75+ years 13.0% in 2014, 14.5% in 2019; *p* < .05), more participants with a degree (15.6% in 2014, 17.8% in 2019; *p <* .05) and more participants were single habiting (17.2% in 2014, 18.4% in 2019; *p <* .05) and less of those with a rural residency (34.6% in 2014, 29.1% in 2019; *p <* .05) in the 2019 sample compared to 2014 (see Table 1). Regarding general health status, 21.3% considered their health to be poor, while 12.9% reported having a mental health problem. Finally, 72.1% had visited a GP within the last 12 months.

**Table 1:** Sample characteristics of *N* = 12,197 participants across two cohorts

	2014 and 2019, %			2014, %			2019, %		
	Total (*N =* 12,197)	Perceived need for treatment	Mental health problem and no psychotherapy	Total (*N =* 6087)	Mental health problem	Mental health problem and no psychotherapy	Total (*N =* 6110)	Mental health problem	Mental health problem and no psychotherapy
Mental health problem	12.9	100.0	100.0	11.8^a^	100.0	100.0	14.0^a^	100.0	100.0
Psychotherapy use									
Psychotherapy	^c^	62.7		^c^	62.8		^c^	62.6	
No psychotherapy	^c^	36.7	100.0	^c^	36.4		^c^	36.9	100.0
No answer	^c^	0.6		^c^	0.8		^c^	0.5	
GP visit	72.1	82.2	84.4	71.4	83.5	85.9	72.8	81.1	83.2
Age, in years									
18–24	7.6	7.1	3.8	8.4^a^	7.7	6.1^d^	6.8^a^	6.6	1.9^d^
25–34	11.8	12.6	11.6	12.2	11.3	8.0	11.4	13.7	14.6
35–44	14.1	15.1	15.5	14.6	14.5	11.9^d^	13.6	15.7	18.4^d^
45–54	19.7	24.0	22.0	20.5^a^	26.6^b^	24.5	18.9^a^	21.8^b^	20.0
55–64	18.4	23.7	24.0	17.2	23.2	24.9	19.6	24.2	23.2
65–74	14.6	8.6	9.7	14.0^a^	8.5	11.1	15.5^a^	8.7	8.6
75+	13.8	8.8	13.4	13.0^a^	8.2	13.4	14.5^a^	9.4	13.3
Gender, women	52.6	64.2	61.6	52.8	65.6	63.6	52.3	63.0	60.0
Education									
School, low	34.8	33.3	37.7	34.0	30.5^b^	35.2	35.6	35.6^b^	39.7
School, middle	32.4	34.9	35.3	33.6	37.5	37.2	31.1	32.8	33.8
School, high	31.9	31.3	26.6	31.7	31.6	27.5	32.1	31.0	25.8
Degree	16.7	14.6	12.5	15.6^a^	14.3	13.0	17.8^a^	14.8	12.1
Single habiting	17.9	23.4	27.9	17.2^a^	23.0	28.6	18.6^a^	23.7	27.3
Health, poor	21.3	40.1	38.1	20.7	40.4	44.7^d^	22.0	39.9	32.7^d^
Rural residency	31.9	28.1	31.9	34.6^a^	29.4	31.7	29.1^a^	27.0	32.1

Weighted percentages (thus, N for percentages is not provided). ^a^ row values of the total sample with the same superscript are significantly different between cohorts at *p <* .05. ^b^ row values of the subsample of those who reported mental health problems with the same superscript are significantly different between cohorts at *p* < .05. ^c^ FILTER Psychotherapy was asked only in those who reported a mental health problem. ^d^ row values of the subsample of those who reported “mental health problems and no psychotherapy” with the same superscript are significantly different between cohorts at *p* < .05

**Fig. 1:**
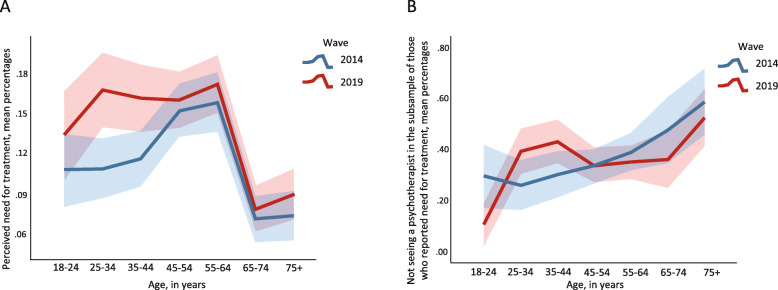
Mean percentages of individuals who reported having a mental health problem that would need to be treated (Panel A) and mean percentage of those not seeing a psychotherapist among those individuals who reported having a mental health problem (Panel B), stratified by age group and by cohort. Weighted cases presented. Confidence intervals 95%. Figure values are provided in Additional file 1

The rate of those reporting a perceived need for treatment was significantly higher in 2019 (14.0%) than in 2014 (11.8%; *p* < .001; Hypothesis 1). For both cohorts, percentages of need for treatment were lowest with 10.8 and 13.5% in 2014 and 2019 in those aged 18–24 and highest in those aged 55–64 (15.9% in 2014 and 17.3% in 2019, respectively). However, in those aged 65–74 (7.1% in 2014, 8.0% in 2019) and 75+ (7.4% in 2014, 9.0% in 2019), the rates of perceived need for treatment were substantially lower than in all younger age groups (see Fig. 1 and Additional file 1).

In those participants who reported a need for treatment, 62.7% saw a psychotherapist, 36.7% did not receive psychotherapy, and 0.6% did not answer this question. Although the absolute number of individuals with a perceived need for treatment increased across cohorts, the proportion of therapy use did not change significantly from 2014 (62.8%) to 2019 (62.6%). Across cohorts by age group, the rate of not seeing a psychotherapist in those with a mental health problem was 19.8% in the cohort aged 18–24 and 37.4% in the 35–44 cohort. Among those aged 65–74, this figure was 41.5%, while among those aged 75+, 55.4% did not see a psychotherapist (see Fig. 1 and Additional file 1).

In order to test the hypothesis of age differences in not receiving or using psychotherapy (Hypothesis 2), the subsample of those who reported a mental health problem that would need assistance was selected for multivariate analyses. In an adjusted model that accounted for the cluster structure of the cohorts and included cohort as a fixed effect (see Table 2), compared to the age group of 18–24 (reference group), those aged 25–34 had an increased probability of OR 2.25 (CI 0.60, 8.43; *p* = .228), those aged 35–44 an OR 2.59 (CI 0.71, 9.42; *p* = 148), and those aged 45–54 an OR 2.12 (CI 0.82, 5.46; *p* = .122) of not receiving psychotherapy. In old age, compared to the age group of 18–24, not receiving or using psychotherapy was increased with OR 2.72 (CI 1.47, 5.05; *p* = .001) in the 65–74 cohort and OR 4.76 (CI 2.09, 10.87; *p* < .001) in the 75+ cohort. The associations of psychotherapy use and age were comparable in a model with and without further confounders (see Table 2).

**Table 2:** Multiple associations with not using psychotherapy in those reporting a mental health problem that would need help

	Unadjusted model				Adjusted model			
	*OR*	*LL CI*	*UL CI*	*p*	*OR*	*LL CI*	*UL CI*	*p*
Cohort, 2019 (Ref. 2014)	0.93	0.92	0.94	<.001	0.94	0.94	0.94	<.001
Age 18–24 (Ref.)	–	–	–	–	–	–	–	–
Age 25–34	2.09	0.60	7.24	.247	2.25	0.60	8.43	.228
Age 35–44	2.42	0.71	8.20	.156	2.59	0.71	9.42	.148
Age 45–54	2.04	0.87	4.76	.100	2.12	0.82	5.46	.122
Age 55–64	2.34	1.15	4.77	.019	2.36	1.02	5.50	.046
Age 65–74	2.80	1.69	4.64	<.001	2.72	1.47	5.05	.001
Age 75+	4.97	2.55	9.69	<.001	4.76	2.09	10.87	<.001
Gender, women					0.83	0.78	0.88	<.001
Education								
School, low (Ref.)					–	–	–	–
School, middle					0.97	0.91	1.04	.363
School, high					0.84	0.76	0.94	.001
Degree					0.86	0.69	1.08	.198
Single habiting					1.37	1.35	1.38	<.001
Health, low					0.75	0.44	1.27	.278
Rural residency					1.38	1.22	1.56	<.001
GP visit					1.39	1.37	1.41	<.001

*N* unweighted = 1666. Weighted coefficients presented. OR = odds ratio. LL CI and UL CI = lower and upper limit 95% confidence interval of the odds ratio coefficient. Logistic Generalized Estimation Equation (GEE) models were specified (weighting factor was used; distribution = binomial; link function = logit; repeated = cohort; type of covariance = exchangeable; robust estimator was used)

Regarding Hypotheses 3, in the adjusted model, not using psychotherapy was associated with single habiting OR 1.37 (1.35, 1.38; *p* < .001) and rural residency (OR 1.38; CI 1.22, 1.56; *p <* .001). Educational attainment was not associated with the use of psychotherapy (although high school education was related with lower probability of not receiving psychotherapy). Women were more likely to receive psychotherapy (OR 0.83; 0.78, 0.88; *p <* .001). In this model, those who reported GP visits (Hypothesis 4) were more likely not to use psychotherapy (OR 1.39; 1.37, 1.41; *p <* .001), which was independently of the overall health status which was accounted for (OR 0.75; 0.44, 1.27; *p* = .278).

## Discussion

The present study provides rare evidence on psychotherapy non-utilization across age groups in two separate nationwide cohorts of German community-dwelling adults with a focus on the development in old age. Main results indicate that the individuals from the cohort of 2014 (Hypothesis 1), older age (Hypothesis 2), male gender, lower educational status, rural residency, single habiting (all Hypothesis 3) and seeing a GP (Hypothesis 4) to be related with non-utilization of psychotherapy. Findings are largely in line with proposed Hypotheses. Only general health status was not significantly associated with non-utilization.

The rate of perceived need for treatment was significantly elevated from about 12 % in 2014 to 14 % in 2019 (Hypothesis 1). Although this effect may be due to an increased awareness of mental health problems in older adults, our results echo recent findings from Germany showing an increase in administrative prevalence of depressive disorders in the outpatient care setting from almost 13 % in 2009 to almost 16 % in 2017; although this effect was largely due to a relative increase in younger cohorts under 24 years of age [13]. This indicated not directly an increased prevalence of mental health problems over time, but an increase in diagnostics and potentially in mental health service use. Further, we found that in those participants, who reported perceived need for treatment, almost two third of the population saw a psychotherapist. Conversely, one-third did not receive treatment by a psychotherapist. In the literature on unmet need for treatment, Nadeem et al. [26] reported a rate perceived need for treatment of 54.7% in a US sample of depressed low-income women; while only 8.2% received mental health treatment. Seeking for treatment was 62.4% of those having a major depression in a study by Mojtabai et al. [19]; 31.9% reported an unmet need for treatment. In a study of subclinical depressed participants, 27% received treatment, 33% reported an unmet need, while 40% had no perceived need and the authors conclude that not subclinical depressed individuals may need help for their depressive symptoms [27].

Regarding the relation of increased age with non-utilization of psychotherapy, there was an increased probability for middle-aged cohorts of not receiving psychotherapy compared with those adults under 26 years of age (Hypothesis 2). In old age, not receiving or using psychotherapy was strongly elevated in the cohort aged 65–74 years and dramatically in those 75+ years of age. This finding is much in line with previous evidence on mental health service research of studies conducted 10 years or more in the past [10, 11, 28, 29]. More recent studies support our findings of higher rates of non-utilization of psychotherapy and lower referral rates in older adults than working-age adults [12, 30]. In addition, Crabb and Hunsley [11], for instance, found that compared to depressive individuals aged 45–64, those people over 65 with depression were less likely to report a mental health consultation in the past year. This held especially true for psychotherapists, as most consultations were with a GP. In Germany, mental health care includes inpatient and outpatient services. Outpatient care is mainly provided by psychotherapists, psychiatrists, specialists in psychosomatic medicine, licensed clinical psychologists, and GPs. Inpatient care is mainly provided at psychiatric hospitals and psychosomatic clinics. To accurately portray the association between perceived need and non-utilization of mental health care, future research should examine the utilization of mental health care services provided by different mental health care professionals, not only psychotherapists.

Despite general awareness about an age-related gap in the non-utilization of mental health services, less is known about its underlying causes. However, past research has identified some relevant factors in the underutilization of mental health services among older adults, such as patients negative stigma and beliefs associated with mental health and mental health care [14, 20], negative self-perceptions of aging [31], personal beliefs of older adults [17], unmet needs [32], lack of professional training and knowledge in geriatrics and aging [33], and lack of organizational structures such as interdisciplinary approaches and collaborative care models [8, 34, 35]. The vastly lower levels of psychotherapy utilization in older adults relative to the younger cohorts are especially concerning. In principle, psychotherapy resources are plentiful, and there are no additional charges for patients within the statutory health insurance system in Germany.

Hypotheses 3: With regard to the role of gender in the utilization of psychotherapy, past research has reported mixed results: while Wei et al. [36] found no gender differences in psychotherapy non-utilization, others reported higher rates in women than in men [17]. Our result endorses gender differences in the utilization of mental health services. Further, the finding is in line with past research acknowledging a series of gender-related barriers in seeking mental health support [37]. Our finding on single habiting may be related to fewer instrumental and emotional spousal and social support resources that foster help-seeking behavior for psychotherapy. In line with previous research, the present study underlines higher non-utilization of mental health services in rural areas [38]. Structural barriers such as the availability of and geographic distance from mental health services may be related to the lower utilization of mental health services in rural areas. Further, we found some evidence about the relationship between higher education and psychotherapy, although the picture was not clear. Using Medicare claims data, Wei et al. found in a sample of older adults with depression, older age, high educational attainment, and the structural unavailability of psychotherapy providers were related to non-utilization of psychotherapy [36].

Those who reported GP visits were more likely not to use psychotherapy, which was independent of the overall health status (Hypothesis 4). This finding is in line with previous research on both the key role of GPs in the treatment of mental health problems [35, 39, 40] and findings on a distinct mental health under-treatment by mean of psychotherapy in the primary care setting [34, 41], especially among older adults [42]. Some research found GPs to provide comprehensive depression treatments for their patients as they are able to take into account the complex comorbidity history of their older patients [43]. Nonetheless, previous findings also proposed a lack of skills and knowledge in mental health among GPs [41, 44, 45]. However, a systematic review and meta-analysis examining the role of GP training in depression care found no improvement in care. Instead, collaborative care models are suggested as a more promising strategy for improving depression care [46]. Strengthening collaborations between GPs and psychotherapists, increasing rates of referrals, and improving diagnostics skills and reimbursement would potentially improve rates of psychotherapy utilization in older adults. Finally, our finding of high rates of psychotherapy non-utilization in older adults is especially unsatisfactory as meta-analytical evidence supports the effectiveness of psychotherapy in older adults, although effectiveness varies largely across studies and samples [7]. In sum, results remain inconclusive. Further research is needed to examine promising approaches to improve diagnostics and treatment of mental health in primary care, especially for older adults.

### Strength and limitations

This study has substantial strengths, including two cohorts of a large and nationwide weighted sample as well as a sample of old and oldest old individuals. Utilizing this sample, we provide needed evidence of the distribution of psychotherapy use across age groups. Limitations of this study include that the perceived need for treatment is a subjective measure and, thus, was self-reported. Self-evaluated need for treatment, per definition, differs from clinical diagnoses. For instance, Grobe et al. [47] found administrative diagnoses within the healthcare system showed higher rates compared with survey self-reported depressive symptoms, which, in turn, may be different from perceived need for treatment [19, 26]. Despite these differences, self-perceptions of mental health problems and need for treatment have their own value as they are close to mental components of subjective wellbeing.

Secondly, we used single items only. However, GP visits and therapy use are suitable for single items. Further, single items to measure mental health have been shown to be correlated with multi-item scales. Thus, future studies should use elaborated scales such as PHQ-9 or HADS to validate our findings [48]. Thirdly, we did not assess comorbidities but used a measure for subjective health status only. Although this measure has advantages such as the feasibility across the whole age range, future studies should look into the outpatient treatment of depression in combination with multimorbidity or specific comorbidities such as dementia [49, 50]. Finally, the non-use of psychotherapeutic services in the case of an existing mental health problem could result from several effects: psychotherapy was advised but not sought out; psychotherapy was not advised; psychotherapy itself was not wanted (for various reasons); psychotherapy was desired but could not be achieved (for a variety of reasons). As our study did not differentiate between these variants, future research should elaborate on factors of non-utilization in more depth. We do not know whether the psychotherapy would have been indicated, whether it was advised, whether it was desired, whether it was not achieved.

### Practical implications and future research

Practical implications of this research include the underpinning of the lack of psychotherapeutic treatment in older cohorts, especially when considering that demographic change will increase this problem. Large proportions of older adults may benefit from effective treatment of psychotherapy [7] yet are likely undertreated. Referring these individuals to psychotherapy – if adequate – could improve mental health and wellbeing of older adults without potential complications that have been shown to be associated with pharmacotherapy [51]. Diagnostic and treatment pathways across healthcare sectors and professions must be optimized to face this challenge. Structural improvements, such as better access to mental health services in rural areas, need to be implemented. Close cooperation between GPs and psychotherapists could also encourage possible psychotherapy use [39]; in particular, collaborative care models may be promising [21, 46]. Besides structural improvements, interdisciplinary training of skills and abilities with regard to diagnosis and therapy of age-related depression should be made available. Building on gender differences in the prevalence of mental health problems and utilization of mental health services, access to mental health services need to be organized in a more specific manner with specific efforts targeting older and male individuals. Further, universities should consider increasing their emphasis on mental health, especially among older adults, in their core and advanced curricula for general practice. We have to acknowledge that most treatment of depression occurs in primary care. Our findings suggest an increased ratio of treatment in primary care rather than psychotherapy as people are older. Treatment of mentally ill patients in primary care may be adequate in individuals with complex multimorbidity patterns. Nonetheless, age stereotypes and inadequate undertreatment related to ageism that further expand this ratio need to be challenged [9, 52, 53].

Besides our primary finding of the gap between the perceived need for treatment and non-utilization, we found a lower perceived need in older people than in younger cohorts. Explanations include internalized age stereotypes (e.g., the false assumption that there is no effective treatment for old age depression or that older people have to cope with depression on their own [9]), but also the fact that the low rate of utilization in this cohort of older adults may lead to a decreased perceived need (i.e., negative feedback loop). Future studies should look into the psychological, sociodemographic, and structural factors that may explain the differences in need perception of older adults compared to younger people [9]. Further, we found differences, for instance, between rural and urban settings. Thus, our findings may also reflect differences in attitudes towards seeking mental health services among people living in rural and non-rural areas, which should be investigated in future research [31]. Finally, in Germany, in addition to psychotherapists, other mental health professionals (for example, psychiatrists) play a key role in the provision of mental healthcare. Future research should examine the association between perceived need and non-utilization and other mental health professionals.

Our study showed that in Germany, psychotherapy non-utilization in those with a perceived need for treatment due to mental health problems was stronger in older adults than in younger ones. Further, non-utilization was associated with GP visits. More research is needed that disentangles the specific mechanisms and patterns between patients, GPs, and psychotherapists that allow for optimal care and care pathways in the treatment of mental health problems for all ages.

## Supplementary Information

**Additional file 1: Table S1.** Percentages of those who reported perceived need for treatment by age category and by cohort. **Table S2.** Percentages of those individuals not seeing a psychotherapist in those reporting a mental health problem by age category and by cohort.

## Data Availability

The datasets generated during and analyzed during the current study will be stored in a non-publically available repository. The access information is available from the PI and the corresponding author on reasonable request. To ensure confidentiality, data dispersed to project team members will be blinded of any identifying participant information.

## References

[1] WHO: Mental health of older adults. Retrieved from https://wwwwhoint/news-room/fact-sheets/detail/mental-health-of-older-adults on Mar 03, 2019 2017, World Health Organisation.

[2] Raue PJ, McGovern AR, Kiosses DN, Sirey JA (2017). Advances in psychotherapy for depressed older adults. Curr Psychiatry Rep.

[3] Huang AX, Delucchi K, Dunn LB, Nelson JC (2015). A systematic review and meta-analysis of psychotherapy for late-life depression. Am J Geriatr Psychiatry.

[4] Wilkinson P, Ruane C, Tempest K (2018). Depression in older adults. BMJ.

[5] Arts MH, Collard RM, Comijs HC, Zuidersma M, de Rooij SE, Naarding P, Oude Voshaar RC (2016). Physical frailty and cognitive functioning in depressed older adults: findings from the NESDO study. J Am Med Dir Assoc.

[6] Heser K, Stein J, Luppa M, Wiese B, Mamone S, Weyerer S, et al. Late-life depressive symptoms are associated with functional impairment cross-sectionally and over time: results of the AgeMooDe study. J Gerontol B Psychol Sci Soc Sci. 2018. 10.1093/geronb/gby083. 10.1093/geronb/gby083 29986090

[7] Kok RM, Reynolds CF (2017). Management of Depression in older adults: a review. JAMA.

[8] Unützer J (2002). Diagnosis and treatment of older adults with depression in primary care. Biol Psychiatry.

[9] Kessler EM, Schneider T (2019). Do treatment attitudes and decisions of psychotherapists-in-training depend on a Patient's age?. J Gerontol B Psychol Sci Soc Sci.

[10] Bogner HR, de Vries HF, Maulik PK, Unützer J (2009). Mental health services use: Baltimore epidemiologic catchment area follow-up. Am J Geriatr Psychiatry.

[11] Crabb R, Hunsley J (2006). Utilization of mental health care services among older adults with depression. J Clin Psychol.

[12] Chaplin R, Farquharson L, Clapp M, Crawford M (2015). Comparison of access, outcomes and experiences of older adults and working age adults in psychological therapy. Int J Geriatr Psychiatry.

[13] Steffen A, Thom J, Jacobi F, Holstiege J, Bätzing J (2020). Trends in prevalence of depression in Germany between 2009 and 2017 based on nationwide ambulatory claims data. J Affect Disord.

[14] Byers AL, Arean PA, Yaffe K (2012). Low use of mental health services among older Americans with mood and anxiety disorders. Psychiatr Serv.

[15] Lavingia R, Jones K, Asghar-Ali AA. A Systematic Review of Barriers Faced by Older Adults in Seeking and Accessing Mental Health Care. J Psychiatr Pract. 2020;26(5):367–82. 10.1097/PRA.0000000000000491 32936584

[16] Andersen RM. National Health Surveys and the Behavioral Model of Health Services Use. Med Care. 2008;46(7):647–653. 10.1097/MLR.0b013e31817a835d 18580382

[17] Volkert J, Andreas S, Harter M, Dehoust MC, Sehner S, Suling A, Ausin B, Canuto A, Crawford MJ, Da Ronch C (2018). Predisposing, enabling, and need factors of service utilization in the elderly with mental health problems. Int Psychogeriatr.

[18] Babitsch B, Gohl D, von Lengerke T (2012). Re-revisiting Andersen's Behavioral Model of Health Services Use: a systematic review of studies from 1998–2011. Psychosoc Med.

[19] Mojtabai R (2009). Unmet need for treatment of major depression in the United States. Psychiatr Serv.

[20] Kammerer K, Falk K, Heintze C, Dopfmer S, Heusinger J (2019). GPs' views on barriers and preconditions for referring elderly people with depressive disorder to psychotherapy. Gesundheitswesen.

[21] Anthony JS (2010). Baik S-y, bowers BJ, Tidjani B, Jacobson CJ, Susman J: conditions that influence a primary care Clinician's decision to refer patients for depression care. Rehabil Nurs.

[22] Nurit G-Y, Dana P, Yuval P (2015). Predictors of psychotherapy use among community-dwelling older adults with depressive symptoms. Clin Gerontol.

[23] Henderson C, Potts L, Robinson EJ (2020). Mental illness stigma after a decade of time to change England: inequalities as targets for further improvement. Eur J Pub Health.

[24] KBV: Insured Survey of the German National Association of Statutory Health Insurance Physicians. https://wwwkbvde/html/versichertenbefragungphp 2014, 2019.

[25] Bullinger M, Kirchberger I (1998). Physical Health Questionnaire - German Version [based on MOS Short-Form-36 Health Survey (SF-36; Ware, J.E., Snow, K.K., Kosinski, M., & Gandek, B., 1993)].

[26] Nadeem E, Lange JM, Miranda J (2009). Perceived need for care among low-income immigrant and US-born black and Latina women with depression. J Women's Health.

[27] van Zoonen K, Kleiboer A, Beekman AT, Smit JH, Boerema AM, Cuijpers P (2015). Reasons and determinants of help-seeking in people with a subclinical depression. J Affect Disord.

[28] Cooper C, Bebbington P, McManus S, Meltzer H, Stewart R, Farrell M, King M, Jenkins R, Livingston G (2010). The treatment of common mental disorders across age groups: results from the 2007 adult psychiatric morbidity survey. J Affect Disord.

[29] Han B, Gfroerer JC, Colpe LJ, Barker PR, Colliver JD (2011). Serious psychological distress and mental health service use among community-dwelling older U.S. adults. Psychiatr Serv.

[30] Pettit S, Qureshi A, Lee W, Stirzaker A, Gibson A, Henley W, Byng R (2017). Variation in referral and access to new psychological therapy services by age: an empirical quantitative study. Br J Gen Pract.

[31] Kessler EM, Agines S, Bowen CE (2015). Attitudes towards seeking mental health services among older adults: personal and contextual correlates. Aging Ment Health.

[32] Stein J, Pabst A, Weyerer S, Werle J, Maier W, Miebach L, Scherer M, Stark A, Kaduszkiewicz H, Wiese B, Moor L, Bock JO, König HH, Riedel-Heller SG (2016). Unmet care needs of the oldest old with late-life depression: a comparison of patient, caring relative and general practitioner perceptions - results of the AgeMooDe study. J Affect Disord.

[33] Karel MJ, Gatz M, Smyer MA (2012). Aging and mental health in the decade ahead: what psychologists need to know. Am Psychol.

[34] Fleury M-J, Imboua A, Aubé D, Farand L, Lambert Y (2012). General practitioners' management of mental disorders: a rewarding practice with considerable obstacles. BMC Fam Pract.

[35] Gonçalves DC, Coelho CM, Byrne GJ (2014). The use of healthcare services for mental health problems by middle-aged and older adults. Arch Gerontol Geriatr.

[36] Wei W, Sambamoorthi U, Olfson M, Walkup JT, Crystal S (2005). Use of psychotherapy for depression in older adults. Am J Psychiatr.

[37] Addis ME, Hoffman E (2017). Men's depression and help-seeking through the lenses of gender.

[38] Brenes GA, Danhauer SC, Lyles MF, Hogan PE, Miller ME (2015). Barriers to mental health treatment in rural older adults. Am J Geriatr Psychiatry.

[39] Gaebel W, Kowitz S, Fritze J, Zielasek J (2013). Use of health care services by people with mental illness: secondary data from three statutory health insurers and the German statutory pension insurance scheme. Dtsch Arztebl Int.

[40] Holvast F, Verhaak PFM, Dekker JH, de Waal MWM, van Marwijk HWJ, Penninx BWJH, Comijs H (2012). Determinants of receiving mental health care for depression in older adults. J Affect Disord.

[41] Trautmann S, Beesdo-Baum K (2017). The treatment of depression in primary care. Dtsch Arztebl Int.

[42] Mitchell AJ, Rao S, Vaze A (2010). Do primary care physicians have particular difficulty identifying late-life depression? A meta-analysis stratified by age. Psychother Psychosom.

[43] Wallace E, Salisbury C, Guthrie B, Lewis C, Fahey T, Smith SM (2015). Managing patients with multimorbidity in primary care. Br Med J.

[44] Brown JD, Wissow LS (2012). Rethinking the mental health treatment skills of primary care staff: a framework for training and research. Adm Policy Ment Health Ment Health Serv Res.

[45] Gilbody S, Whitty P, Grimshaw J, Thomas R (2003). Educational and organizational interventions to improve the management of depression in primary care: a systematic review. Jama.

[46] Sikorski C, Luppa M, König H-H, van den Bussche H, Riedel-Heller SG (2012). Does GP training in depression care affect patient outcome? - a systematic review and meta-analysis. BMC Health Serv Res.

[47] Grobe TG, Kleine-Budde K, Bramesfeld A, Thom J, Bretschneider J, Hapke U (2019). Prävalenzen von Depressionen bei Erwachsenen–eine vergleichende Analyse bundesweiter Survey-und Routinedaten. Das Gesundheitswesen.

[48] May T, Pridmore S (2019). A visual analogue scale companion for the six-item Hamilton depression rating scale. Aust Psychol.

[49] Gellert P, Häusler A, Gholami M, Rapp M, Kuhlmey A, Nordheim J. Own and partners’ dyadic coping and depressive symptoms in individuals with early-stage dementia and their caregiving partners. Aging Ment Health. 2017;22(8):1008–16. 10.1080/13607863.2017.1334759. 10.1080/13607863.2017.1334759 28594233

[50] Nordheim J, Häusler A, Yasar S, Suhr R, O’Sullivan J, Kuhlmey A, et al. Psychosocial intervention in couples coping with dementia: the DYADEM trial;2019;68(2):745–55. 10.3233/JAD-180812. 10.3233/JAD-180812 30775982

[51] Coupland C, Dhiman P, Morriss R, Arthur A, Barton G, Hippisley-Cox J (2011). Antidepressant use and risk of adverse outcomes in older people: population based cohort study. BMJ.

[52] Cuijpers P, Karyotaki E, Weitz E, Andersson G, Hollon SD, van Straten A (2014). The effects of psychotherapies for major depression in adults on remission, recovery and improvement: a meta-analysis. J Affect Disord.

[53] Kessler EM, Blachetta C (2020). Age cues in patients' descriptions influence treatment attitudes. Aging Ment Health.

