# Supplementary material for: Perceived need for treatment and non-utilization of outpatient psychotherapy in old age: two cohorts of a nationwide survey
Source: BMC Health Serv Res. 2021 May 10;21:442. doi: 10.1186/s12913-021-06384-6 (PMC8111709; doi:10.1186/s12913-021-06384-6)
Supplement: Supplementary file 1 — Additional file 1: Table S1. Percentages of those who reported perceived need for treatment by age category and by cohort. Table S2. Percentages of those individuals not seeing a psychotherapist in those reporting a mental health problem by age category and by cohort. [file 12913_2021_6384_MOESM1_ESM.docx]

**Perceived need for treatment and non-utilization of outpatient psychotherapy in old age: Two cohorts of a nationwide survey**

Paul Gellert^1^

Sonia Lech^1^

Eva-Marie Kessler^2^

Wolfram Herrmann^3^

Susanne Döpfmer^3^

Klaus Balke^4^

Monika Oedekoven^1^

Adelheid Kuhlmey^1^

Susanne Schnitzer^1^

*^1^ Charité – Universitätsmedizin Berlin, Institute of Medical Sociology and Rehabilitation Science, Berlin, Germany*

*^2^ MSB Medical School Berlin, Gerontopsychotherapy, Berlin, Germany*

*^3^ Charité – Universitätsmedizin Berlin, Institute of General Practice, Berlin, Germany*

*^4^ German National Association of Statutory Health Insurance Physicians (Kassenärztliche Bundesvereinigung KBV), Berlin, Germany*

*Corresponding author:* Paul Gellert, Charité – Universitätsmedizin Berlin, Charitéplatz 1, 10117 Berlin, Germany, [paul.gellert@charite.de](mailto:paul.gellert@charite.de)

Additional file 1.

Table S1. Percentages of those who reported perceived need for treatment by age category and by cohort.

| Perceived need for treatment | Age category | | | | | | |  |
| --- | --- | --- | --- | --- | --- | --- | --- | --- |
|  | 18-24 | 25-34 | 35-44 | 45-54 | 55-64 | 65-74 | 75+ | Total |
| 2014, % | 10.8^a^ | 10.9^a^ | 11.7^a^ | 15.3^a^ | 15.9^a^ | 7.1^a^ | 7.4^a^ | 11.8 |
| 2019; % | 13.5^a^ | 16.8^b^ | 16.1^b^ | 16.1^a^ | 17.3^a^ | 8.0^a^ | 9.0^a^ | 14.0 |
| 2014 and 2019; % | 12.0 | 13.7 | 13.8 | 15.7 | 16.6 | 7.6 | 8.3 | 12.9 |

Note. Percentages in one column with the same superscript are not statistically different at p <.05. Percentages relate to Figure 1 Panel A.

Table S2. Percentages of those individuals not seeing a psychotherapist in those reporting a mental health problem by age category and by cohort.

| No psychotherapy | Age category | | | | | | |  |
| --- | --- | --- | --- | --- | --- | --- | --- | --- |
|  | 18-24 | 25-34 | 35-44 | 45-54 | 55-64 | 65-74 | 75+ | Total |
| 2014, % | 29.1^a^ | 25.9^a^ | 29.8^a^ | 33.5^a^ | 39.2^ab^ | 47.5^ab^ | 59.3^b^ | 36.4 |
| 2019; % | 10.7^a^ | 39.3^b^ | 43.3^b^ | 34.1^b^ | 35.3^b^ | 36.5^b^ | 52.5^b^ | 36.9 |
| 2014 and 2019; % | 19.8^a^ | 33.8^ab^ | 37.4^b^ | 33.8^ab^ | 37.0^b^ | 41.5^bc^ | 55.4^c^ | 36.7 |

Note. Percentages in one column with the same superscript are not statistically different at p <.05. Percentages relate to Figure 1 Panel B.
